# Diverse unsaturated fatty acids bypass loss of FabH-catalysed initiation of fatty acid synthesis in Enterococcus faecalis

**DOI:** 10.1099/mic.0.001740

**Published:** 2026-07-15

**Authors:** Qi Zou, Huijuan Dong, John E. Cronan

**Affiliations:** 1 Department of Microbiology, University of Illinois at Urbana Champaign, Urbana, Illinois, USA; 2 Department of Biochemistry, University of Illinois at Urbana-Champaign, Urbana, Illinois, USA

**Keywords:** fatty acid synthesis, initiation reaction, phospholipid synthesis

## Abstract

The initiation reaction of fatty acid synthesis in *Enterococcus faecalis* is catalysed by the FabH 3-ketoacyl-acyl carrier protein (ACP) synthase III. Deletion of the *fabH* gene seriously impaired growth but failed to fully block *de novo* fatty acid synthesis. We report that exogenous medium-chain unsaturated fatty acids restore normal growth of a *∆fabH* strain and upon elongation provide substrates for synthesis of functional membrane phospholipid bilayers. Entry of these acids into the elongation pathway was aided by increased expression of the AcpA ACP of fatty acid synthesis and/or of the phosphate: ACP acyltransferase PlsX. The efficacy of incorporation of these acids increased with increasing chain length. Coordinate overexpression of *E. faecalis* AcpA and PlsX or expression of the *Lactococcus lactis* PlsX allowed elongation of the short-chain octanoic acid to provide unsaturated fatty acids for growth of the *∆fabH* strain. We also tested the abilities of the two long-chain β-ketoacyl-ACP synthases, FabO and FabF, in elongation of exogenous unsaturated fatty acids.

## Introduction

**Fig. 1.**
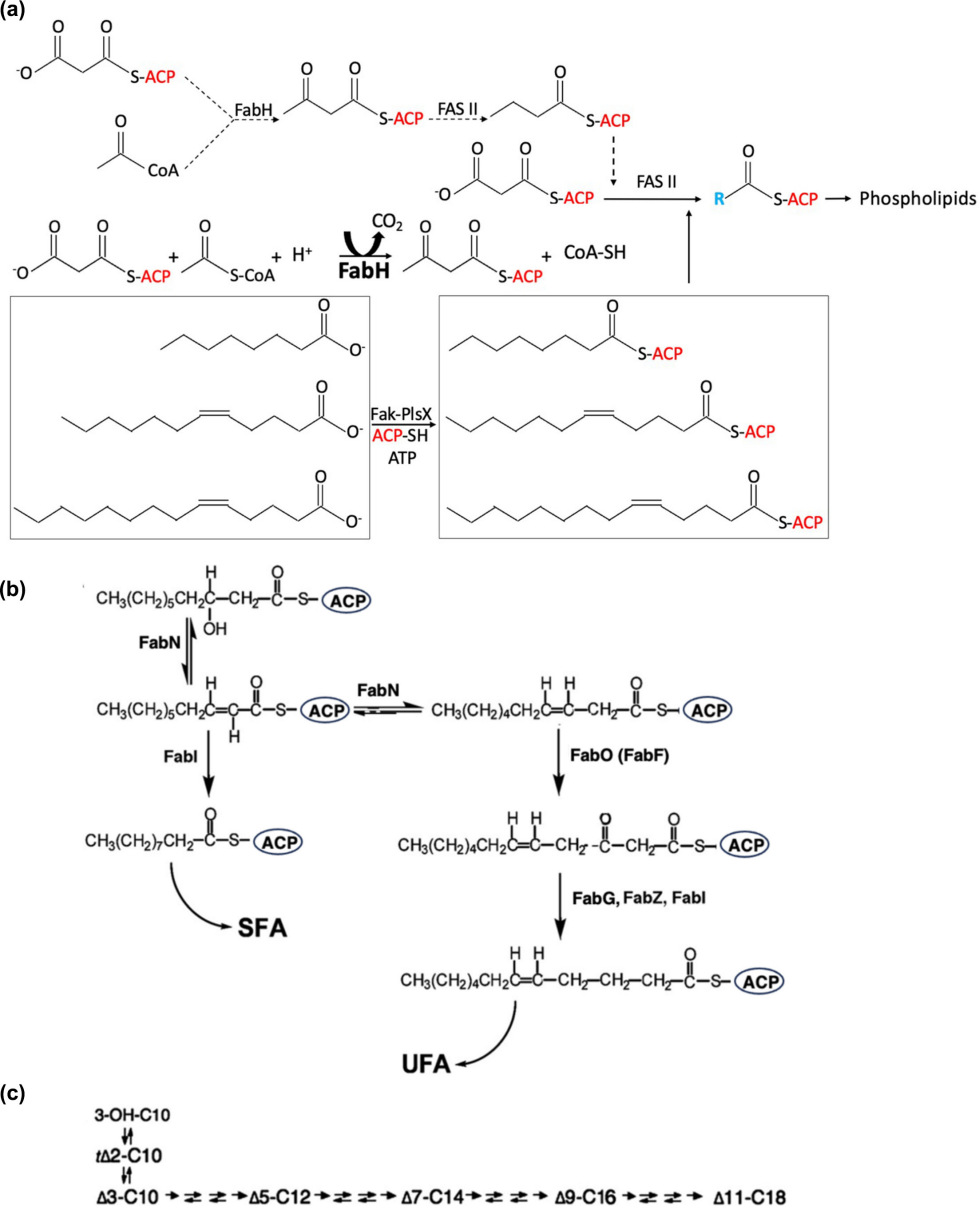
**(a)** Elongation of exogenous short-chain fatty acids by *E. faecalis* FAS II for phospholipid acyl chains. In the FabH reaction shown in the upper left of panel a, the top compound is malonyl-ACP and that immediately below is acetyl-CoA. The FabH reaction is shown in more detail immediately below the schematic. The acyl carrier protein ACP is labelled in red. R denotes the long carbon chains produced from *de novo* synthesis or exogenous incorporation. The blue arrow represents the growing acyl chains. (**b)** The reactions of unsaturated acyl-ACP synthesis in *E. faecalis*. The pathway parallels that of *Escherichia coli* with FabN in place of FabA and FabO in place of FabB with the exception that *E. faecalis* FabF has some activity in the FabO reaction. (**c)** Carbon flow in the unsaturated acyl chain synthesis pathway [24]. The arrows between each intermediate denote, in order, the 3-ketoacyl-ACP synthase (FabO or FabF), 3-ketoacyl-ACP reductase (FabG), the 3-hydroxyacyl-ACP dehydratase (FabZ) and the enoyl-ACP reductase (FabI). The 3-ketoacyl-ACP synthase reaction is irreversible due to decarboxylation. Although the FabI reaction is reversible on paper, it is irreversible in practice because rupture of the two kinetically stable C–H bonds formed upon reduction requires a more positive reduction potential than those of NAD+ or NADP+. In contrast, the FabG and FabZ reactions are reversible.

 Fatty acid biosynthesis is a ubiquitous bacterial metabolic pathway that provides precursors for cell membrane phospholipid bilayer synthesis, as well as for coenzymes, signalling molecules and protein post-translational modification [1,2]. This process generally utilizes the type II fatty acid synthesis (FASII) pathway, a set of discrete enzymes found in bacteria, mitochondria and plant plastids. The FabH 3-ketoacyl-acyl carrier protein (ACP) synthase III (KAS III) is a highly conserved FASII component that catalyses the initiation reaction of the pathway. FabH elongates acetyl-CoA by condensation with malonyl-ACP to produce short-chain 3-ketoacyl-ACPs (C4, C6). Following their conversion to butyryl- or hexanoyl-ACPs, these act as primers for the *Enterococcus faecalis* long-chain 3-ketoacyl-ACP synthases, FabO and FabF in synthesis of the C16 and C18 phospholipid acyl chains (Fig. 1abc) [3,4].

As discovered by Rock and coworkers in Firmicute bacteria such as *E. faecalis* [5,8], exogenous fatty acids are activated by the two-component fatty acid kinase system (FakA/B) to form acyl-phosphates. The acyl-phosphates can either be directly utilized by the G3P acyltransferase PlsY to acylate the *sn*-1 position of glycerol 3-phosphate to form lysophosphatic acid or be converted to acyl-ACPs by the phosphate acyltransferase PlsX for acylation of the *sn*-2 position of lysophosphatidic acid by PlsC to synthesize the key phospholipid synthesis intermediate, phosphatic acid [8]. If the exogenous acids are too short to be incorporated into phospholipids, the acyl-ACPs derivatives can enter the fatty acid synthesis cycle and be elongated to the C16 and C18 chains required for phospholipid synthesis [9].

**Fig. 2.**
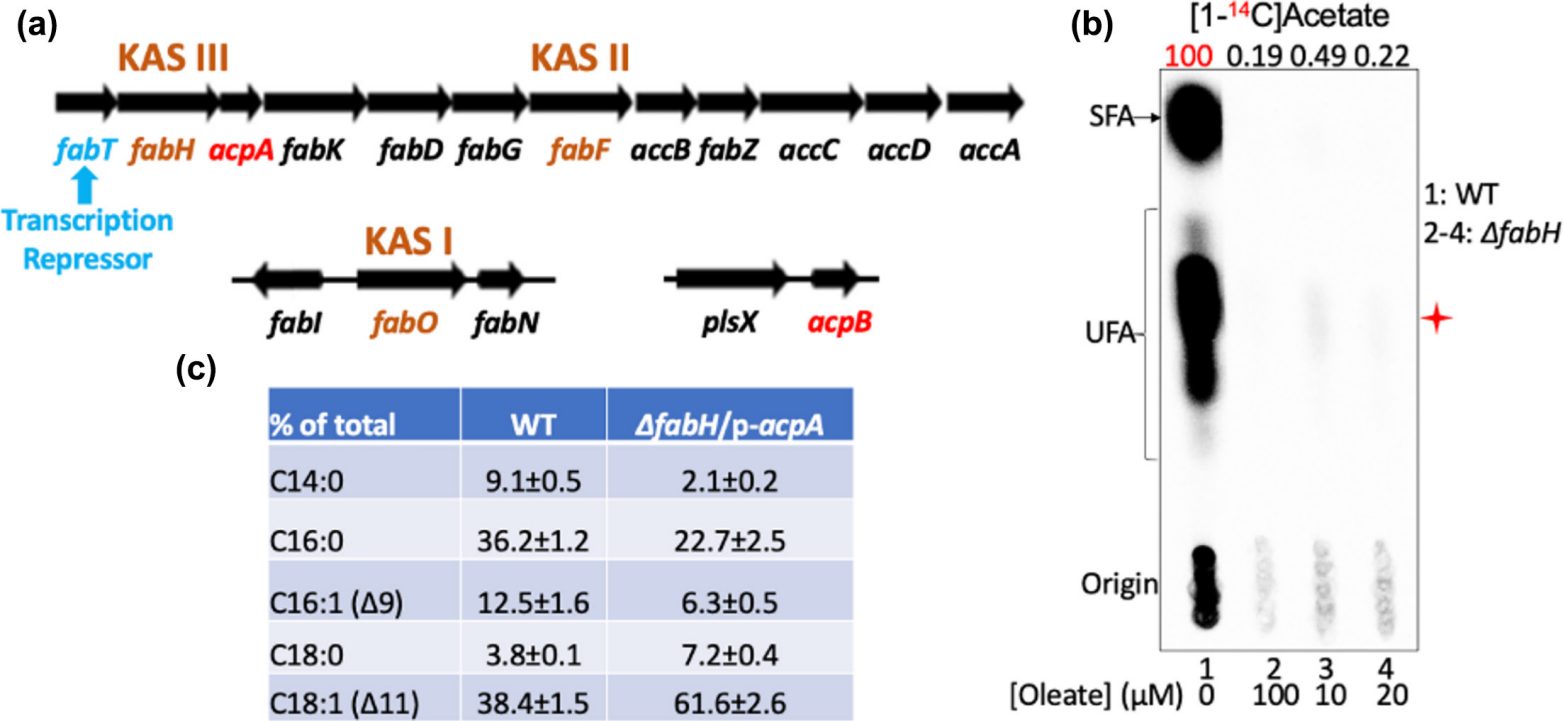
Fatty acid synthesis is not fully blocked in the *E. faecalis ∆fabH* strain. (**a)** *E. faecalis* genome arrangement of fatty acid metabolism genes. The *fabT* gene is labelled in blue, the two *acp* genes are in red, and the three KAS genes are labelled in brown. (**b)** *De novo* fatty acid synthesis *of E. faecalis ∆fabH* strain in the presence of 10 or 20 µM oleic acid added to support growth. The 100 µM in lane 2 was included to demonstrate full repression of the pathway and the lesser repression given by lower concentrations. The numbers above the lanes are the radioactive label incorporation values relative to the value (100) for the wild-type (WT) strain cultured without exogenous fatty acids. The red star highlights the *de novo* synthesized radiolabelled fatty acyl chains in the *∆fabH* strain. (**c)** GC–MS analysis of phospholipid acyl chain composition of the *E. faecalis ∆fabH* strain with AcpA-overexpression and the WT strain.

*E. faecalis* is a Gram-positive, facultative anaerobic, opportunistic pathogen with high levels of antibiotic resistance [10]. The bacterium inhabits the gastrointestinal tract of humans and other animals and is the cause of many hospital-acquired infections [10]. Unusual for bacteria, *E. faecalis* encodes two acyl carrier proteins: AcpA, responsible for *de novo* fatty acid synthesis [11], and AcpB, which functions in extracellular free fatty acid incorporation and pathway regulation [12,13]. This bacterium contains three 3-ketoacyl-acyl carrier protein synthases. FabH (KAS III) initiates the synthesis of acyl chains as discussed below, whereas FabO (KAS I) is largely responsible for unsaturated fatty acid synthesis and FabF (KAS II) catalyses elongation to produce long-chain fatty acyl species with modest activity in unsaturated fatty acid synthesis [3] (Fig. 2a). FabF is also required for temperature-controlled synthesis of *cis*-vaccenic acid [14].

*E. faecalis* and other members of the order Lactobacillales avidly incorporate exogenous fatty acids into their membrane phospholipids [15]. Indeed, exogenous long-chain unsaturated fatty acids can almost completely replace *de novo* synthesized acyl chains in membrane phospholipids [9,12, 16]. This is due to increased FabT repression of the *de novo* pathway by binding of acyl-ACPs derived from long-chain exogenous acids [12,13]. In the wild-type (WT) *E. faecalis* strain, we previously reported that upon conversion to acyl-AcpA species, short- and medium-chain saturated fatty acids enter fatty acid synthesis and become elongated to phospholipid acyl chains [9]. The question was whether the flux through the pathway is sufficient for these acids to bypass the initiation reaction catalysed by FabH. As first seen in *Lactococcus lactis* [17] and as shown below in *E. faecalis, ∆fabH* strains lacking FabH (KAS III) are long-chain fatty acid auxotrophs, as are other null mutants in the *E. faecalis* fatty acid synthesis pathway [3,11, 18, 19]. However, relief of auxotrophy in these strains requires that the long-chain acids (C16, C18) are unsaturated species; saturated fatty acids are inactive and can inhibit growth [16]. This restriction means that bypass of the *∆fabH* phenotype cannot be tested by elongation of short- or medium-chain saturated fatty acids. However, a test can be performed with medium-chain unsaturated acids, and we report such experiments here. An advantage of *∆fabH* strains over other fatty acid synthesis null mutants is that, excepting initiation, fatty acid synthesis remains intact. In a WT strain, the weak or absent FabT repression exerted by short-chain acids [16] means that these acids must compete with *de novo* synthesis for elongation and incorporation into phospholipids. Hence, competition from *de novo* synthesis may obscure utilization of an exogenous acid. In the case of unsaturated acids too short for direct incorporation into phospholipids, the derived acyl-ACP must both bypass loss of FabH and become elongated to a functional phospholipid acyl chain. Can medium-chain unsaturated acids bypass the *∆fabH* strain initiation deficiency? We tested this using non-native unsaturated fatty acids that are readily distinguished from the native acyl chains. A C16 acid, *cis*-7-hexadecenoic acid, relieved *∆fabH* strain auxotrophy as did a C14 acid, *cis*-5-tetradecenoic acid, but the shorter C12 acid, *cis*-5-dodecenoic acid had weaker activity. We also tested if the short-chain acids, hexanoic (C6) and octanoic acid (C8) can be sufficiently elongated to unsaturated phospholipid acyl chains to bypass auxotrophy of a *∆fabH* strain. Finally, we probed the division of labour between the two *E. faecalis* long-chain 3-ketoacyl-acyl carrier protein synthases, FabO and FabF, to test if both enzymes are proficient in elongation of unsaturated acyl chains.

## Methods

### Materials

Cayman Chemicals provided the *cis*-5-tetradecenoic acid and all the other fatty acids, ortho-nitrophenyl-β-galactoside, and antibiotics were purchased from Sigma-Aldrich. The M17 broth, tryptone and yeast extract (Becton Dickenson) were purchased from Thermo Fisher Scientific. The DNA polymerase, restriction endonuclease, T4 DNA ligase and Gibson Assembly Cloning Kit were purchased from New England Biolabs. Sodium[1-^14^C]acetate (specific activity, 57.0 mCi mmol^−1^) and sodium[1-^14^C]octanoate (specific activity, 55.0 mCi mmol^−1^) were supplied by Moravek, Inc. Silver nitrate-silica gel thin-layer plates were purchased from Analtech. All the other reagents were of the highest available quality. Oligonucleotide primers were synthesized by Integrated DNA Technologies and DNA sequencing was performed by ACGT, Inc.

### Bacterial strains and plasmids

The bacterial strains and plasmids used in this study are listed in Table S1, available in the online Supplementary Material. *E. faecalis* cultures were grown at 37℃ in M17 medium. Erythromycin was added at 5 mg l^−1^. Incubation times are given in the figure legends.

### Growth of *E. faecalis* strains

To examine growth of *E. faecalis*, the strains were streaked on M17 plates containing 1.5% agarose with or without an exogenous fatty acid at the concentration given. Incubation times at 37℃ are given in the figure legends. Precultures of the strains requiring an unsaturated fatty acid were grown in M17 medium with oleic acid or *cis*-vaccenic acid.

### TLC analysis of radioactively labelled fatty acid methyl esters from bacterial phospholipids

The TLC assay for radiolabelled phospholipid acyl chains was described previously [9,13]. To test synthesis of phospholipid fatty acyl chains, 5 ml *E. faecalis* cultures were inoculated at OD_600_ of 0.1 in M17 medium and incubated at 37℃ for 6 h in the presence of 1 mCi l^−1^ sodium [1-^14^C]acetate with or without a single exogenous fatty acid at the concentration described above. The cells were lysed with methanol/chloroform (2 : 1) solution and the phospholipids were extracted by chloroform and then dried under nitrogen. The fatty acyl chains were transesterified by treatment with 25% (wt/vol.) sodium methoxide, extracted into hexanes and processed for TLC analysis on Analtech silica gel containing 20% silver nitrate in toluene at −20 °C. The TLC plates containing the [^14^C]-labelled fatty acid methyl esters were exposed and quantitated by phosphorimaging on a GE Typhoon FLA700 scanner, and the data were analysed using ImageQuant TL software.

To test incorporation of octanoic acid (C8 : 0), *E. faecalis* strains were inoculated at OD_600_ of 0.1 in 5 ml of M17 medium, respectively, containing 0.1 mCi l^−1^ sodium [1-^14^C]octanoate plus 1 mM non-radioactive octanoic acid and cultured at 37°C for 6 h. The fatty acyl chains on phospholipids were extracted, methylated and analysed as described above.

### GC–MS analysis of phospholipid acyl chains

To analyse the fatty acyl chains of bacterial phospholipids, *E. faecalis* strains were inoculated at an OD_600_ of 0.1 in M17 medium with or without the presence of a single fatty acid at the concentration illustrated above at 37°C for 6 h. The conversion of phospholipids to fatty acid methyl esters was the same method used for TLC analysis above and the extracted products were sent for GC–MS analysis. The data were collected in triplicate by Dr. Alexander Ulav of the Metabolic Section of the Carver Biotechnology Center).

### β-Galactosidase assay

The *lacZ* reporter plasmids expressing *Escherichia coli* β-galactosidase from promoters of *E. faecalis fabT* gene (the first gene of *fab* operon, see Fig. 2a) were constructed in the previous work (Zou et al., 2022). The measurement of β-galactosidase activity was processed as previously discussed (Zou et al., 2023). Briefly, *E. faecalis* strains transformed with lacZ reporter plasmids above were cultured to mid-log phase at 37°C, and the harvested cells were washed by PBS, resuspended in Z buffer (60 mM Na_2_HPO_4_, 40 mM NaH_2_PO_4_, 10 mM KCl, 1 mM MgSO_4_), lysed with SDS/chloroform and assayed for β-galactosidase activity. The data were collected in triplicate.

## Results

### The *E. faecalis ∆fabH* strain retains a minimal level of acyl chain synthesis

The loss of the β-ketoacyl-ACP synthase III (KAS III) almost entirely blocked the growth of *E. faecalis* without affecting the expression of the other genes of the *fab* regulon. Faint but visible [^14^C]acetate incorporation into phospholipid acyl chains was seen in the *∆fabH* strain cultured with 10 or 20 µM oleic acid (added to support growth) (Fig. 2b). Thus, severely limited *de novo* synthesis of phospholipid acyl chains remained in the *∆fabH* strain. AcpA overexpression improved growth of the *E. faecalis ∆fabH* strain given long-term incubations and increased the level of *cis*-vaccenic acid (C18 ∆11) in phospholipids (Figs 2c and S2), indicating that AcpA levels were limiting in the *∆fabH* strain (see below). Increased AcpA levels could act by stimulating the production of malonyl-ACP, the acyl-chain building block, because the FadD reaction is reversible and increased ACP will counter the equilibrium of the reaction, which lies towards malonyl-CoA (Fig. 1b). Note that high phospholipid *cis*-vaccenic acid contents are characteristic of *E. faecalis* [20,21].

### Exogenous medium-chain unsaturated fatty acids bypass the initiation step of fatty acid synthesis in *E. faecalis*

Prior work showed that the long-chain unsaturated fatty acids oleic acid (C18 : 1∆9) or palmitoleic acid (C16 : 1∆9) restored growth of *E. faecalis* strains in which *de novo* synthesis gene *acpA* had been deleted although the latter acid was toxic at high concentrations [11,16]. A similar discrimination between the two unsaturated acids was reported in *Staphylococcus aureus* [22]. Bypass of initiation was also seen in a ∆*fabN* strain which lacks the enzyme required for insertion of acyl chain double bonds [3,11, 16] (Fig. 1b). The palmitoleate isomer, *cis*-7-hexadecenoic acid, gave only weak growth of the ∆*fabN* relative to the WT strain [9]. However, the shorter chain unsaturated acids, *cis*-5-tetradecenoic acid (C14:1∆5) or *cis*-5-dodecenoic acid (C12 : 1∆5), were unable to support detectable growth of the ∆*fabN* strain [9].

**Fig. 3.**
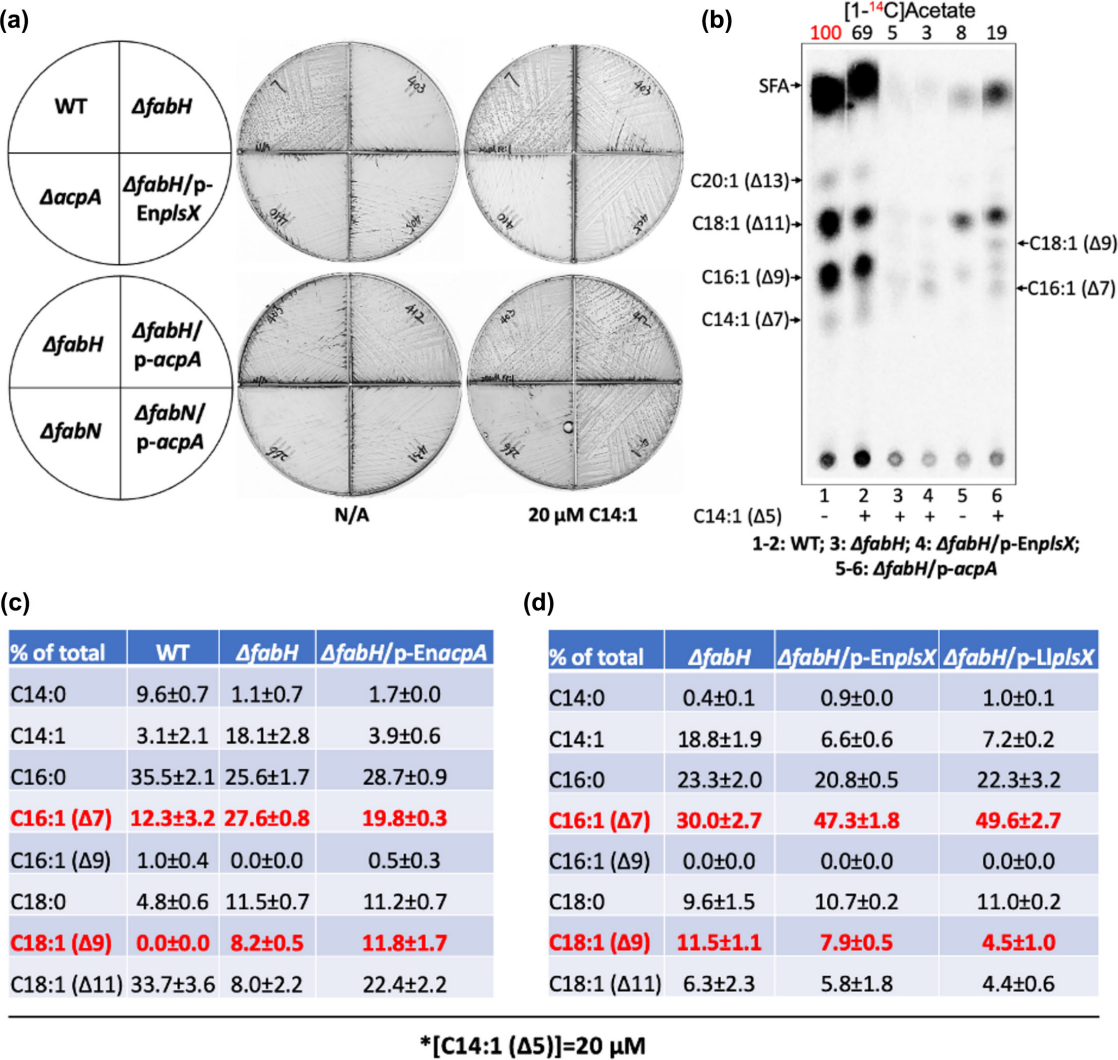
Supplementation with *cis*-5-tetradecenoic acid (C14:1∆5) bypassed the initial reaction of fatty acid synthesis in *E. faecalis*. (a) Growth of the *E. faecalis* ∆fabH strain with or without overexpression of AcpA or PlsX. in the presence of *cis*-5-tetradecenoic acid on M17 medium with overnight incubation. (b) Synthesis of phospholipid fatty acyl chains by the *E. faecalis* ∆fabH strain with or without AcpA or PlsX overexpression in the presence of 20 μM *cis*-5-tetradecenoic acid. The numbers above the lanes are the radioactive label incorporation values relative to the value (100) for the WT strain cultured without exogenous fatty acids. (c and d) GC–MS analysis of the incorporation and elongation products (red font) of *cis*-5-tetradecenoic acid by the *E. faecalis* ∆fabH strain with (c) or without (d) overexpression of AcpA or PlsX. Note that elongation of medium-chain fatty acids shows reduced incorporation of [1-14C]acetate since the phospholipid acyl chains contain fewer labelled acetate units. Hence, the incorporation seen in lane 6 of panel (b) may approximate that of the WT strain depending on the chain lengths of the saturated species that were elongated. WT denotes the WT strain that contained the vector plasmid.

The *∆fabH* strain unlike *∆fabN* or *∆acpA* strains [3,11] grew with 20 µM *cis*-5-tetradecenoic acid (C14 : 1) Fig. 3a and growth was increased by *acpA* overexpression (Fig. 3a). The lack of AcpA in *∆acpA* strains would block elongation of *cis*-5-tetradecenoic acid to the C16, C18 lengths required for synthesis of functional phospholipids. However, in the *∆fabH* strain, the intact cycle of fatty acid synthesis allowed elongation to proceed. The apparent disagreement between the phenotypes of the *∆fabN* and *∆fabH* strains is explained by the high-level synthesis of saturated acyl chains in the *∆fabN* strain but not in the *∆fabH* strain [3]. In *E. faecalis,* incorporation of saturated chains into phospholipids in the absence of unsaturated chains is toxic [16]. Another difference is the residual unsaturated chain synthesis of the *∆fabH* strain which may provide a functional phospholipid membrane until exogenous chains can be elongated.

**Fig. 4.**
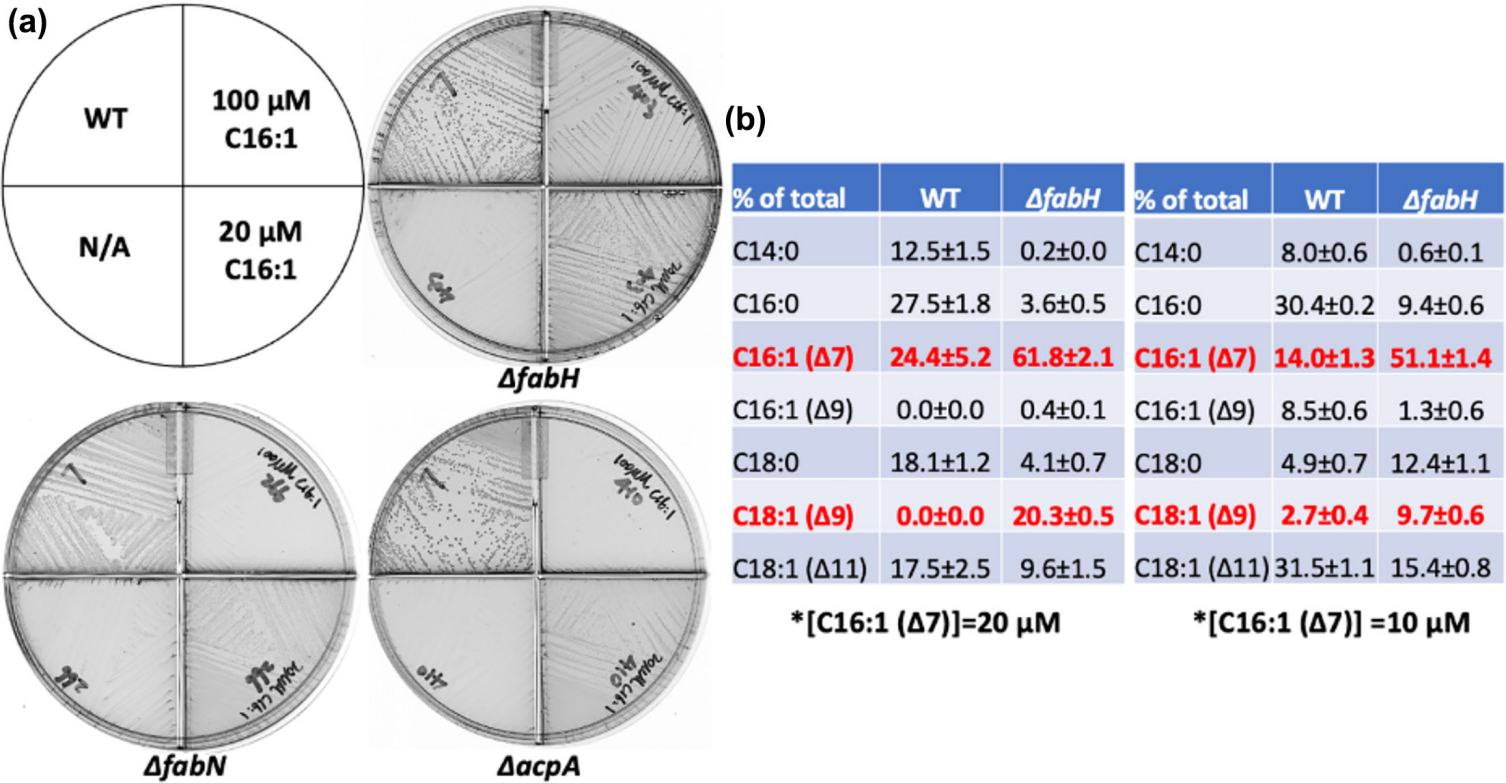
Initiation of fatty acid synthesis in *E. faecalis* is bypassed by palmitoleic acid (*cis*-9-hexadecenoic acid). (**a)** Growth of the *E. faecalis ∆fabH* strain in the presence of palmitoleic acid on M17 agarose medium with overnight incubation. Note that 100 µM palmitoleic acid is toxic, whereas the same concentration of oleic acid is not. (**b)** GC–MS assayed elongation of *cis*-7-hexadecenoic acid by the *E. faecalis ∆fabH* strain. WT denotes the WT strain. N/A denotes no fatty acid addition. The 100 µM palmitoleic acid sector was included to demonstrate its toxicity to *E. faecalis*. Like *E. faecalis, S. aureus* grows well with oleic acid, whereas palmitoleic acid is toxic [7]. No FA was supplied to the WT strain to provide an internal standard. The C19 : 1 cyclopropane acids were combined with the value for their precursor C18 : 1 since cyclopropane synthesis in this bacterium is feeble (a few % of the total acyl chains) and *cfa* deletion strains have no phenotype *in vivo* [31] or *in vitro* [3].

When supplied with *cis*-5-tetradecenoic acid (20 µM) and [1-^14^C]acetate, radioactive C16 : 1(∆7) and C18 : 1(∆9) phospholipid acyl chains were produced, providing a direct demonstration of elongation (Fig. 3b). This was the case in the *E. faecalis ∆fabH* strain with or without overexpression of PlsX or AcpA. GC–MS analysis of the phospholipid acyl chains confirmed elongation of *cis*-5-tetradecenoic acid (Fig. 3c and d). Consistent with the above results, the *∆fabH* strain that overexpressed *acpA* incorporated [1-^14^C]acetate at ~8% of the level of the WT strain in the absence of exogenous fatty acid supplementation with most of the incorporated [1-^14^C]acetate appearing in *cis*-vaccenic acid (C18 ∆11) (Fig. 3b). In the *∆fabH* strain, expression of *L. lactis* PlsX and overexpression of *E. faecalis* PlsX gave comparable increases in incorporation and elongation of *cis*-5-tetradecenoic (Fig. 3d). As expected, the *∆fabH* strain grew upon supplementation with 20 µM palmitoleic acid. This was also true for *cis*-7-hexadecenoic acid, an appreciable portion of which was elongated to oleic acid (Fig. 4a and b). Note that concentrations of palmitoleic acid or *cis*-5-tetradecenoic acid above 20 µM are toxic to *E. faecalis* [9,13].

**Fig. 5.**
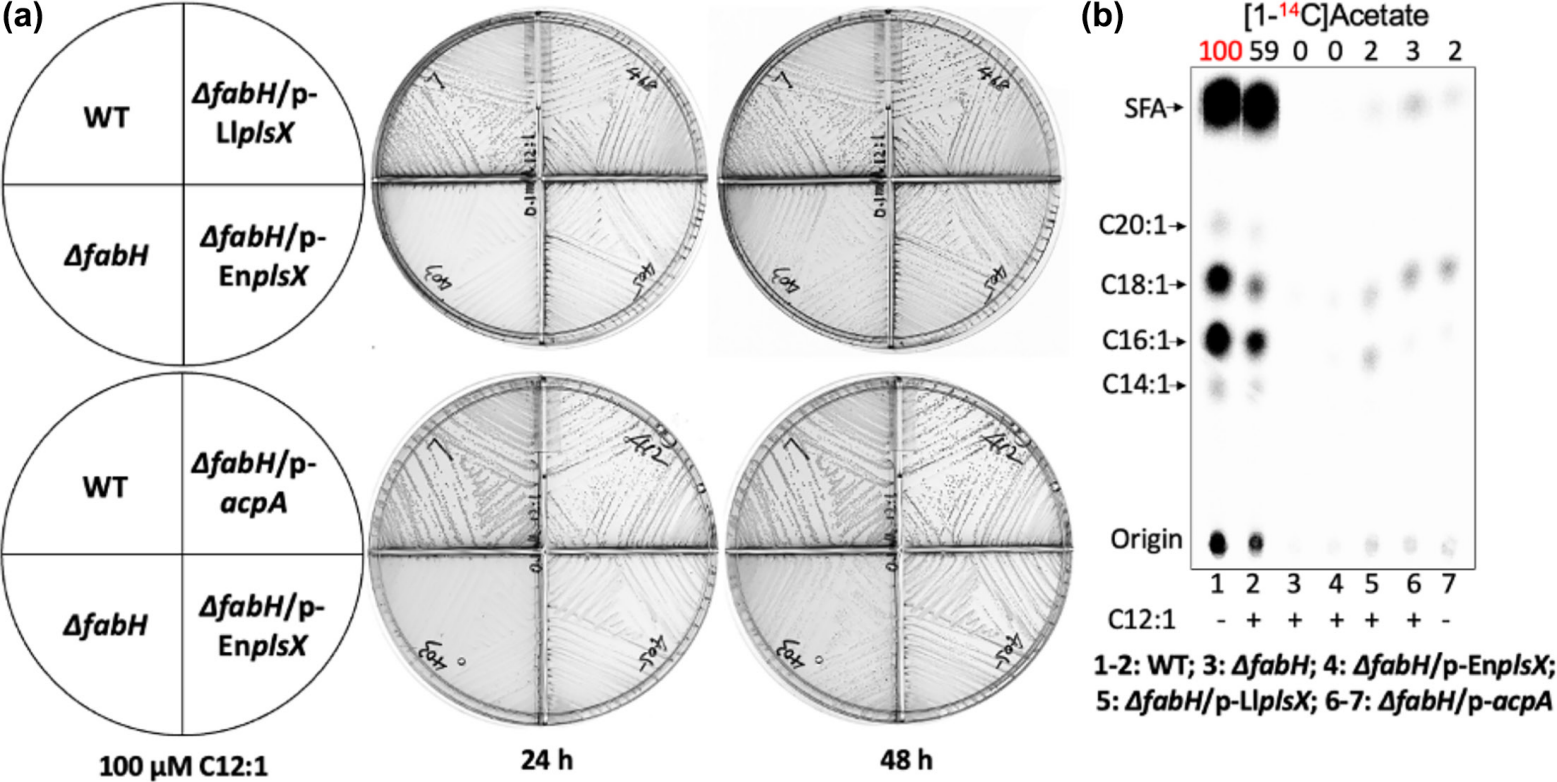
Supplementation with 100 μM *cis*-5-dodecenoic acid (C12:1) was able to bypass initiation of fatty acid synthesis in *E. faecalis*. (a) Effects of improved expression of PlsX or AcpA on the growth of *E. faecalis* ∆fabH strain in the presence or absence of *cis*-5-dodecenoic acid on M17 medium. (b) Synthesis of phospholipid fatty acyl chains by the *E. faecalis* ∆fabH strain with increased expression of PlsX or AcpA in the presence of 100 μM *cis*-5-dodecenoic acid. The numbers above the lanes are the radioactive label incorporation values relative to the value (100) for the WT strain cultured without exogenous fatty acids

**Fig. 6.**
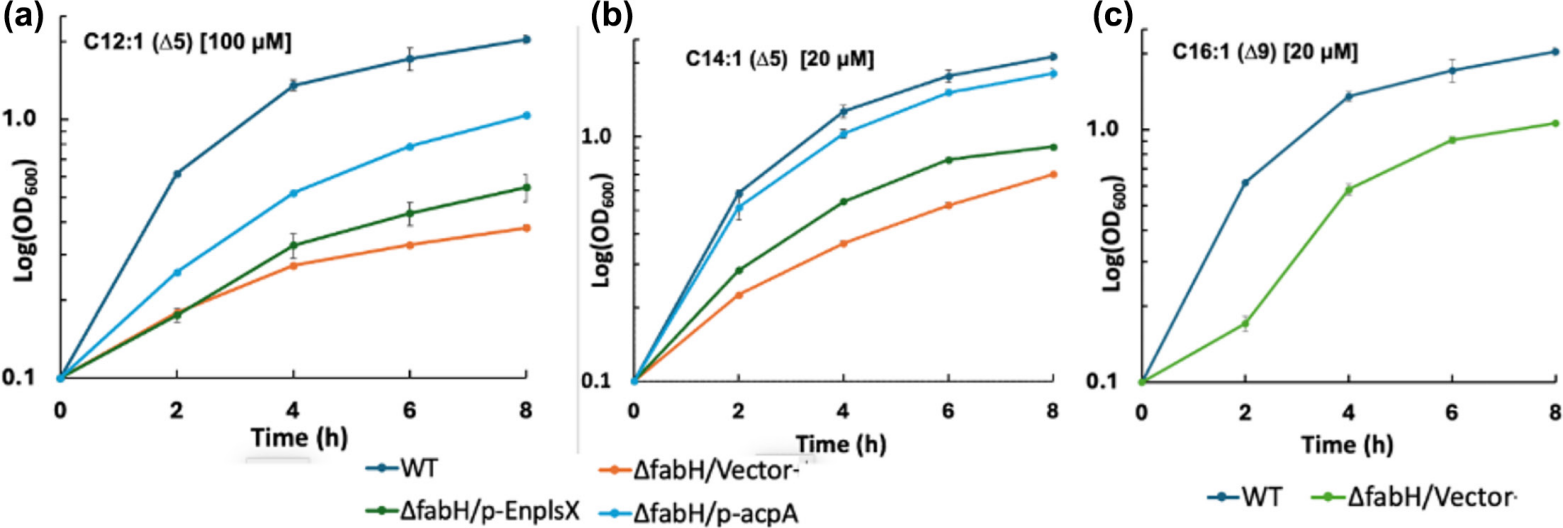
Growth curves in M17 medium containing the unsaturated fatty acid denoted on the panel. The curve identifications of panels (a) and (b) are the same. Panel (c) is growth with palmitoleic acid.

Growth of *E. faecalis ∆fabH* strain was poor when cultured with 20 µM *cis*-5-dodecenoic acid (the concentration used with the longer acids). An increase in concentration to 100 µM of the non-toxic *cis*-5-dodecenoic acid gave detectable growth, although lengthy incubations were required to form discrete colonies (Fig. 5a). These data indicated that the native *E. faecalis* Fak-PlsX system was unable to provide efficient entry of *cis*-5-dodecenoic acid into the FAS pathway even with augmentation of the pathway proteins. This is consistent with the minimal [1-^14^C]acetate incorporation in the presence or absence of *cis*-5-dodecenoic acid (Fig. 5b). Growth curves of the above strains with supplementation on the unsaturated acids are given in Fig. 6.

### High level expression of key proteins allowed octanoate supplementation to bypass lack of FabH

Previous work in the *E. faecalis* WT and *∆fabT* strains showed that [1-^14^C]octanoate was incorporated into both unsaturated and saturated phospholipid acyl chains but only when high levels of both AcpA and *L. lactis* PlsX were present [9]. Overexpression of the cognate PlsX was inferior to expression of the *L. lactis* protein. However, since the *de novo* synthesis pathway was intact in the strains tested previously, the ability of octanoate supplementation to bypass loss of FabH was unknown.

**Fig. 7.**
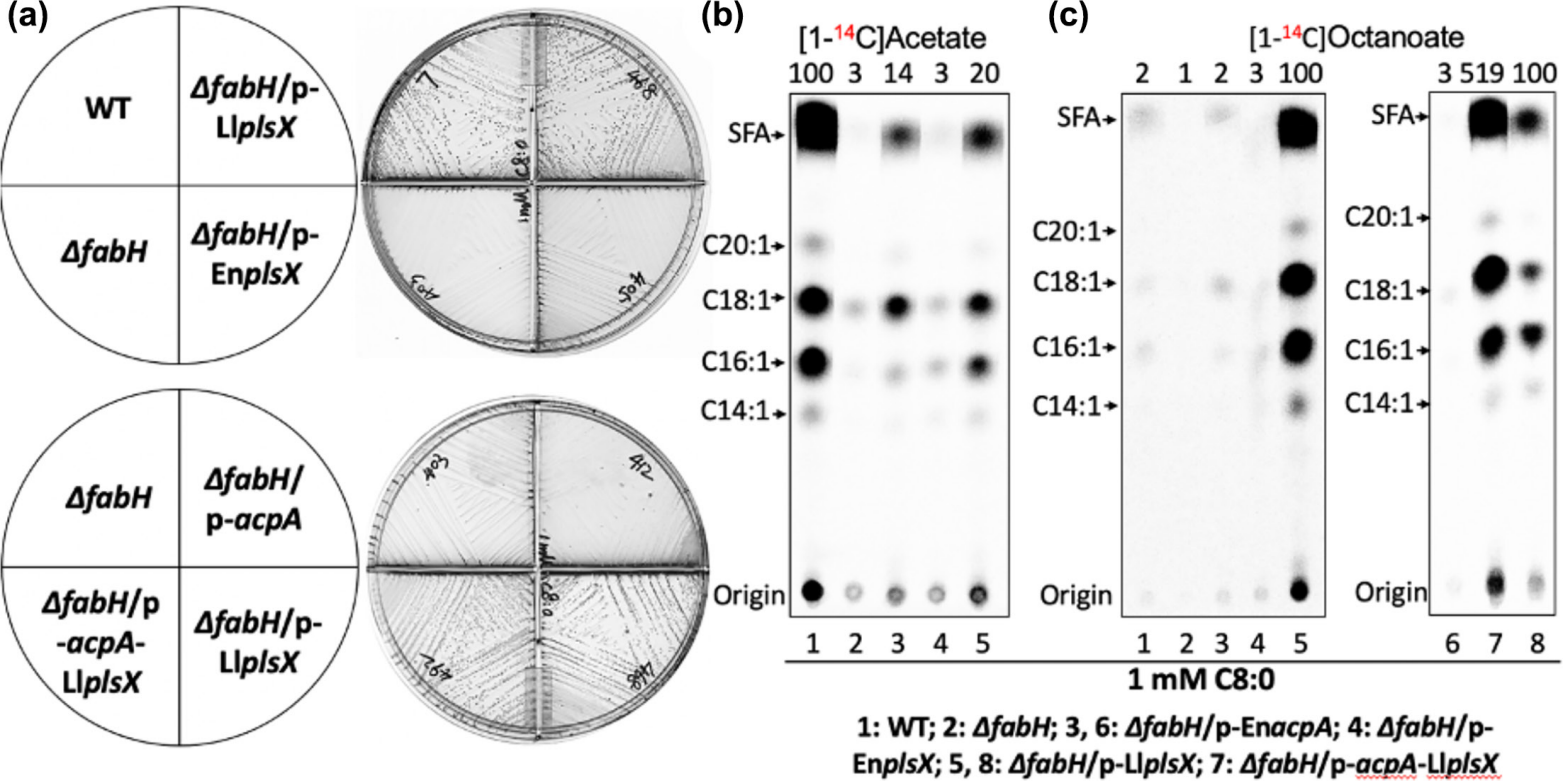
Octanoic acid (C8 : 0) combined with expression of *L. lactis* PlsX permitted *E. faecalis* to bypass the initiation reaction of fatty acid synthesis. (**a)** Growth of the *E. faecalis ∆fabH* strain expressing *L. lactis* PlsX in the presence of C8 : 0 on M17 medium with overnight incubation. (**b)** Synthesis of phospholipid fatty acyl chains by the *E. faecalis ∆fabH* strain with expression of *L. lactis* PlsX in the presence of octanoic acid. The numbers above the lanes are the radioactive label incorporation values relative to the value (100) for the WT strain. (**c)** Effects of expressing *L. lactis* PlsX on incorporation and elongation of [1-^14^C]octanoic acid by the *E. faecalis ∆fabH* strain. The numbers above the lanes are the radioactive label incorporation values relative to the value (100) for the *∆fabH* strain expressing only *L. lactis* PlsX.

Expression of *L. lactis* PlsX in the *E. faecalis ∆fabH* strain resulted in an ~50-fold increase in incorporation of [1-^14^C]octanoate into phospholipid acyl chains relative to the WT strain (Fig. 7c) [16]. An additional fivefold increase in [1-^14^C]octanoate incorporation in the *∆fabH* strain resulted when AcpA was simultaneously overexpressed (Fig. 7c). These manipulations allowed octanoate supplementation to bypass the initiation defect of the *∆fabH* strain by forming an octanoyl-ACP primer to synthesize unsaturated acyl chains which gave improved growth of the *∆fabH* strain (Fig. 6a and b). We also report that overexpression of AcpA plus *L. lactis* PlsX expression allowed hexanoic acid (C6 : 0) to be elongated in the *E. faecalis ∆fabH* strain, and increased growth without supplementation of exogenous fatty acids was seen but only when given 48 h of incubation (Figs S5 and S6). Although overexpression of neither AcpA nor the cognate PlsX alone could allow *E. faecalis* to overcome the *∆fabH* strain initiation deficiency in the presence of octanoic acid (Fig. S3A), this was achieved by coexpression of the two proteins, which resulted in an ~14-fold increase in [1-^14^C]octanoate incorporation to form the phospholipid unsaturated fatty acyl chains required for growth of the *∆fabH* strain (Fig. S3).

**Fig. 8.**
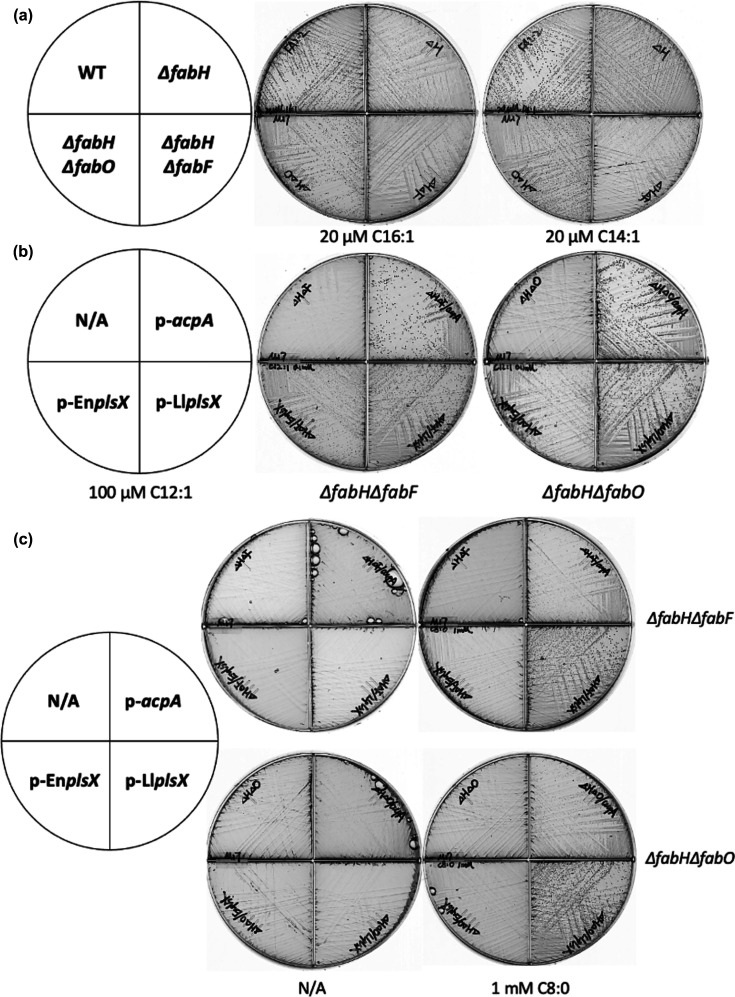
Both *E. faecalis* KAS I (FabO) and KAS II (FabF) independently elongate exogenous short fatty acids to allow growth. (**a)** Growth of *E. faecalis ∆fabH ∆fabF* and *∆fabH ∆fabO* strains in the presence of palmitoleic acid (*cis*-9 C16 : 1) or *cis*-5-tetradecenoic acid on M17 medium incubated overnight. (**b)** Growth of *E. faecalis ∆fabH ∆fabF* or *∆fabH ∆fabO* strains with increased expression of AcpA or PlsX in the presence of *cis*-5-dodecenoic acid on M17 medium with overnight incubation. (**c)** Growth of the *E. faecalis ∆fabH ∆fabF* or *∆fabH ∆fabO* strains with expression of *L. lactis* PlsX in the presence of octanoic acid (C8 : 0) on M17 medium with overnight incubation. N/A denotes no plasmid. The *∆fabO* and *∆fabF* alleles were described previously [3].

Construction of *∆fabH* strains that lacked a long-chain 3-ketoacyl-ACP synthase elongation enzyme, either FabO or FabF, allowed comparison of their abilities to elongate medium-chain unsaturated acids. Both FabO and FabF proved competent in catalysing these elongations (Fig. 8a and b). As expected, elongation of short-chain octanoic acid could be catalysed by either FabO or FabF alone, although FabO was superior to FabF in this process (Figs 7c and S4). Note that loss of *fabF* resulted in a higher proportion of unsaturated phospholipid acyl chains [3,16].

## Discussion

Supplementation with monounsaturated fatty acids bypasses loss of fatty acid synthesis initiation by the paradigm FabH 3-ketoacyl-ACP synthase III in *E. faecalis*. Of the acids tested only palmitoleic acid (C16 : 1∆9) is a native component of *E. faecalis* phospholipids. The other acids were chosen because their double bond positions and/or chain lengths allowed incorporation of the acid and elongation products to be detected by argentation TLC and/or GC–MS analysis of phospholipid acyl chains. Studies of mutant strains blocked in acyl chain synthesis or in the PlsX catalysed interconversion of acyl-phosphates and acyl-ACPs are unsaturated fatty acid auxotrophs, whereas saturated fatty acids are inactive in supporting growth and can inhibit growth [16]. Although *E. faecalis* lacks β-oxidation, its commensal hosts have vigorous oxidation pathways that could produce these acids from dietary sources. The abilities of these acids to bypass loss of FabH activity provide a caution to the efficacy of FabH inhibitors as antibacterial agents for enterococcal and streptococcal infections as first noted in 2009 [23]. *S. aureus* seems an exception because, unlike enterococci and streptococci, this bacterium requires synthesis of branched chain acids which are not abundant in mammals [2].

The almost total lack of *de novo* fatty acid synthesis in the *E. faecalis ∆fabH* strain allows exogenous fatty acid incorporation into phospholipids to be studied without competition from *de novo* synthesis. Moreover, the acyl chain synthesis cycle remains intact allowing chain elongation of exogenous acids to be studied in isolation from *de novo* synthesis, a marked improvement over our prior studies. However, the need for functional *E. faecalis* phospholipids limits investigations in *∆fabH* strains to exogenous unsaturated fatty acids and those short-chain acids that can be efficiently elongated to unsaturated species. Three exogenous unsaturated acids, the native palmitoleate plus the non-native *cis*-7-hexadecenoate and *cis*-5-tetradecenoate, together with the elongation products produced by either FabO or FabF (Fig. 1b) were efficiently incorporated into the phospholipids of the *∆fabH* strain. A surprise was the weaker utilization of *cis*-5-dodecenoate, given that it is a known intermediate in unsaturated acyl chain synthesis [24] (Fig. 1c). However, when tested in a coupled *in vitro* system that assessed acyl-ACP formation from the free acid via FakAB and PlsX, *cis*-5-dodecenoate was as active as *cis*-5-tetradecenoate [9]. However, these were endpoint assays and hence lacked kinetics. A complication in such assays is that ATP hydrolysis by FakAB may push acyl-ACP synthesis such that any kinetic bottlenecks are overcome. Moreover, these assays were necessarily done using the readily expressed and purified AcpB rather than the very recalcitrant AcpA. This is a shortcoming since AcpA is the form required for acyl chain synthesis, whereas AcpB is inactive [11]. Note that given abundant pure AcpA, kinetic analysis would be very challenging due to the number of proteins involved (FakA, four FakBs, PlsX and two ACPs) plus the reversibility of PlsX and the liability of acyl-phosphates and acyl-ACPs.

Given efficient entry of the short-chain acids, the C6 hexanoic acid and the C8 octanoic acid, into the fatty acid synthesis cycle of the *∆fabH* strain, we expected that both acids would be converted to the unsaturated acyl chains required for growth. This is because the derived acyl-ACPs would feed into the fatty acid synthesis cycle prior to the unsaturated/saturated branch point (Fig. 1b). However, our prior work showed that incorporation of these acids required expression of *L. lactis* PlsX and was aided by AcpA overexpression [9]. This was also the case in the *∆fabH* strain. When the prior experiments were repeated in the *∆fabH* strain, growth and long-chain acyl chain synthesis was observed demonstrating efficient bypass of the *∆fabH* phenotype. As previously discussed in detail [9], the superior activity of *L. lactis* PlsX in utilization of these acids can be attributed to the ancient use of *L. lactis* to produce dairy products from milk, a rich source of short- and medium-chain fatty acids.

Loss of *E. faecalis* FabH function engendered auxotrophy for unsaturated fatty acids. However, unlike other *E. faecalis fab* deletion mutants*, ∆fabH* strains retain some acyl chain synthetic activity indicating another initiation pathway(s) is present in this bacterium (Fig. 2). Prior work showed that a *∆fabH* strain of *L. lactis* also retained a low level of *de novo* fatty acid synthesis [17]. *L. lactis* has a stripped-down genome compared to that of *E. faecalis* (30% smaller genome), and lacks AcpB, FabN and FabK [25]. The candidates for FabH-independent initiation are side reactions of long-chain 3-ketoacyl-ACP synthases known to produce an acetyl-ACP primer. These are decarboxylation of malonyl-ACP [26,27] and transfer of the acetyl moiety from acetyl-CoA to the ACP thiol [28]. A distant possibility is a freestanding malonyl-ACP decarboxylase such as that of *E. coli* [29] and other proteobacteria [30]. However, there is no genomic or enzymatic evidence for this reaction in Firmicutes. On the other hand, malonyl-ACP is an intrinsically unstable compound [29] hence decarboxylation would be an undemanding enzyme reaction that could be acquired by a dissimilar protein.

## Supplementary material

10.1099/mic.0.001740 Supplementary Material 1.

## Abbreviations

ACP: acyl carrier protein
FASII: type II fatty acid synthesis
GC: gas chromatography
KAS: 3-ketoacyl-ACP synthase
TLC: thin layer chromatography
WT: wild type
