# Supplementary material for: Diverse unsaturated fatty acids bypass loss of FabH-catalysed initiation of fatty acid synthesis in Enterococcus faecalis
Source: Microbiology (Reading). 2026 Jul 15;172(7):001740. doi: 10.1099/mic.0.001740 (PMC13372179; doi:10.1099/mic.0.001740)
Supplement: Supplementary Material 1. [file mic-172-01740-s001.pdf]

Supplement to

Diverse unsaturated fatty acids bypass the initiation of fatty acid biosynthesis of *Enterococcus faecalis*

Qi Zou, Huijuan Dong and John E. Cronan

Table S1 Strains and Plasmids

Strains and Plasmids	Description	Source
**Strains**		
E. faecalis FA2-2	Wild Type	Lab Stock
E. faecalis DHJ484	Δ*acpA*	Dong & Cronan, 2022a
E. faecalis DHJ497	Δ*fabN*	Dong & Cronan, 2022b
E. faecalis DHJ640	Δ*fabH*	Dong & Cronan In preparation
E. faecalis QZ412	Δ*fabH* with *E. faecalis acpA* expression plasmid	This work
E. faecalis QZ431	Δ*fabN* with *E. faecalis acpA* expression plasmid	This work
E. faecalis QZ405	Δ*fabH* with *E. faecalis plsX* expression plasmid	This work
E. faecalis QZ468	Δ*fabH* with *L. lactis plsX* expression plasmid	This work
E. faecalis QZ477	Δ*fabH* with co-expression plasmid of *E. faecalis acpA* and *E. faecalis plsX*	This work
E. faecalis QZ492	Δ*fabH* with co-expression plasmid of *E. faecalis acpA* and *L. lactis plsX*	This work
E. faecalis QZ104	Δ*fabH* with *L. lactis acpA* expression plasmid	This work
E. faecalis QZ704	Δ*fabH* with *E. coli acpP* expression plasmid	This work
E. faecalis DHJ661	Δ*fabH* Δ*fabF*	Dong & Cronan In preparation
E. faecalis DHJ1056	Δ*fabH* Δ*fabO*	Dong & Cronan In preparation
E. faecalis QZ706	Δ*fabH* Δ*fabO* with *E. faecalis acpA* expression plasmid	This work

E. faecalis QZ707	Δ*fabH* Δ*fabO* with *L. lactis plsX* expression plasmid	This work
E. faecalis QZ708	Δ*fabH* Δ*fabO* with *E. faecalis plsX* expression plasmid	This work
E. faecalis QZ709	Δ*fabH* Δ*fabF* with *E. faecalis acpA* expression plasmid	This work
E. faecalis QZ710	Δ*fabH* Δ*fabF* with *L. lactis plsX* expression plasmid	This work
E. faecalis QZ711	Δ*fabH* Δ*fabF* with *E. faecalis plsX* expression plasmid	This work
E. faecalis QZ219	FA2-2 with *lacZ* expression plasmid from *fabT* promoter	Zou et al., 2022
E. faecalis QZ541	Δ*fabH* with *lacZ* expression plasmid from *fabT* promoter	This work
**Plasmid**		
pQZ28	Shuttle plasmid vector with a p32 promoter modified from pZL277 by replacement of chloramphenicol resistance with erythromycin resistance, *E. faecalis* expression	Zou et al., 2023
pQZ43	*E. faecalis plsX* in pQZ28	Zou et al., 2023
pDHJ544	*L. lactis plsX* in pQZ28	Zou et al., 2024
pQZ31	*E. faecalis acpA* in pQZ28	Zou et al., 2024
pQZ472	pQZ28 with *acpA* and *E. faecalis plsX*	Zou et al., 2024
pQZ490	pQZ28 with *acpA* and *L. lactis plsX*	Zou et al., 2024
pQZ214	*E. faecalis fabT* start region (-389 to +35) at 5'-end of *lacZ* in pBHK322	Zou et al., 2022

Note that for the "in preparation strains" the *ΔfabO* and *ΔfabF* alleles were reported previously (Dong & Cronan, 2022b). The primers used to construct the *ΔfabH* allele are given in Table S2.

Table S2 Oligonucleotides primers used in the study

**Primers***	**Sequence 5'-3'**
EcacpP NcoI F	CATGCCATGGCTATGAGCACTATCGAAGAACG
EcacpP EcoRI R	CCGGAATTCTTACGCCTGGTGGCC
Ef fabH up2	GAATTAGTTGTACAAGCCGTGTTGAAAATGGGACC TTAAT
Ef fabH dn2	TAGAACTAGTAGGGATCCCCCGGGCTGCAGTTTTA CGACTTCTTTTGGG

*The underlined sequences indicate the restriction sites used in this work.

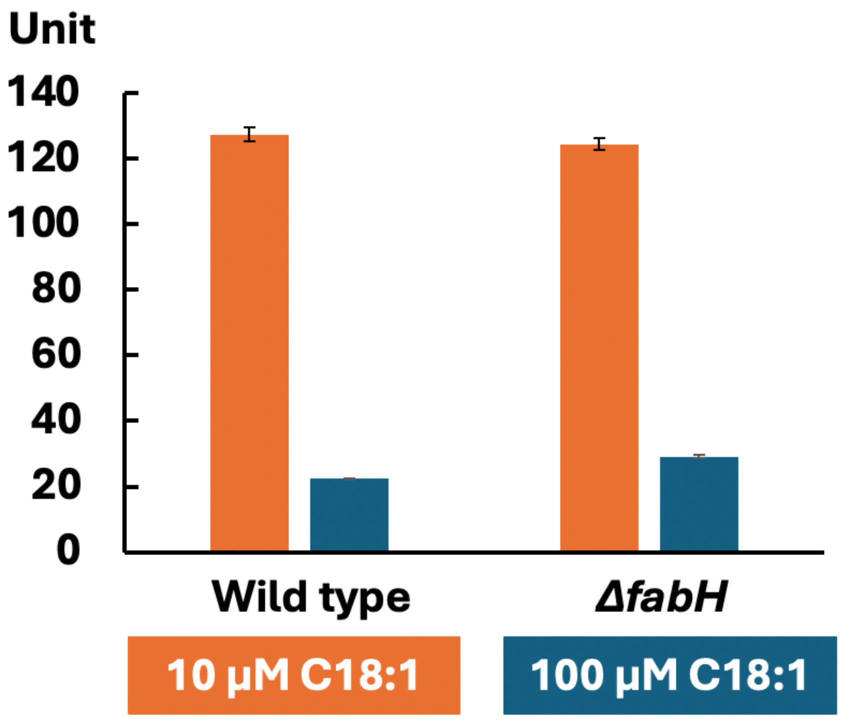

Fig. S1. Deletion of the *fabH* gene did not alter expression or regulation of the *E. faecalis* fatty acid synthesis regulon. The effects of oleic acid on expression of β-galactosidase driven by the *E. faecalis fabT* promoter. Oleic acid supplementation was required to support growth of the Δ*fabH* strain.

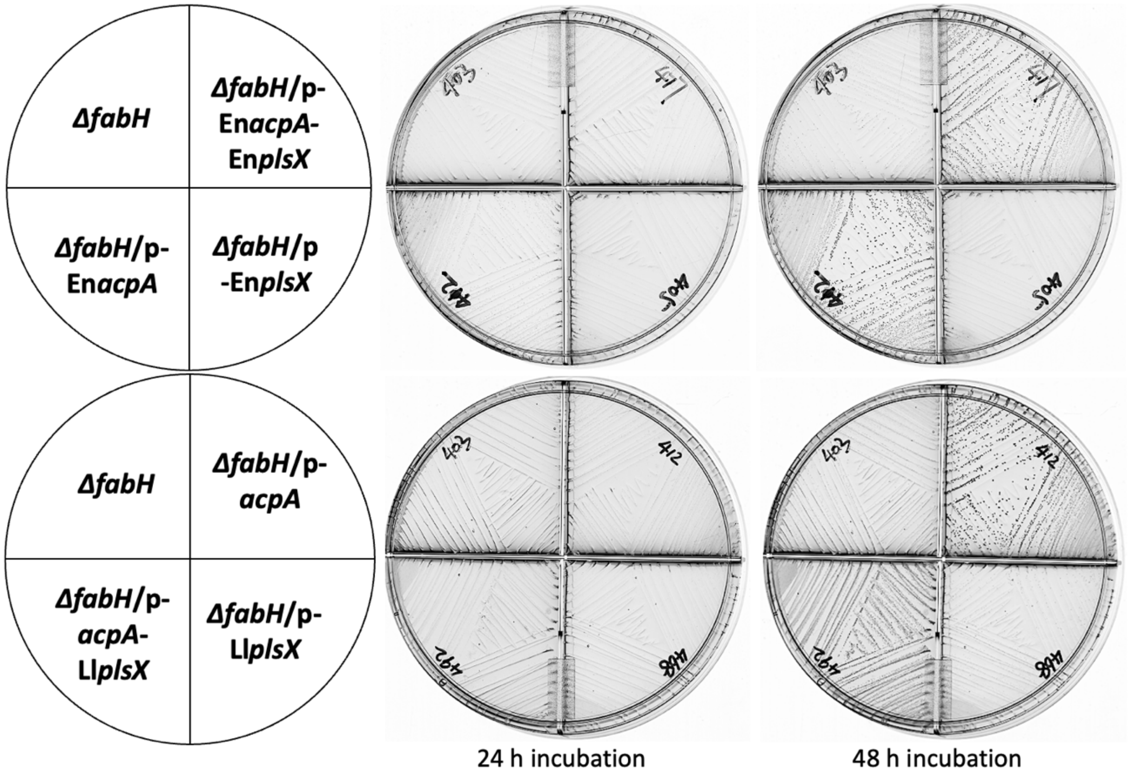

Figure S2. Overexpression of AcpA restored growth of the *E. faecalis* Δ*fabH* strain given long-term incubation. The *E. faecalis* strains were streaked on M17 medium lacking exogenous fatty acid supplementation.

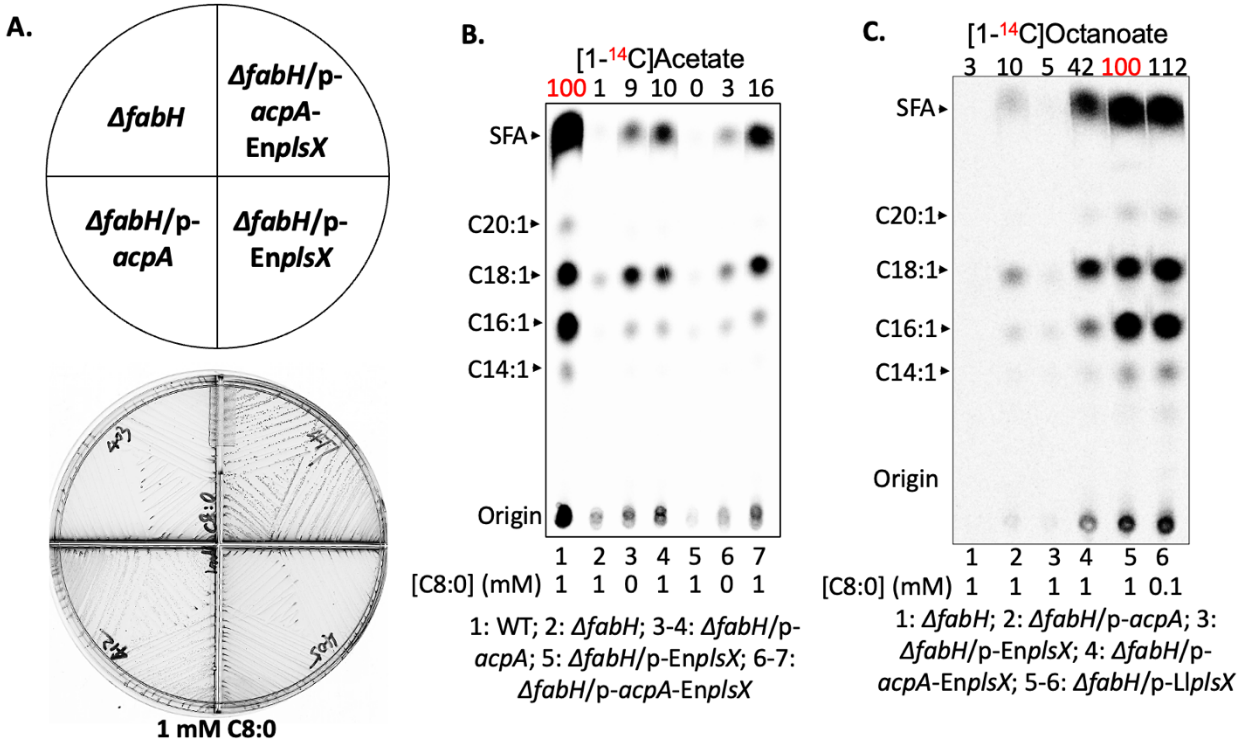

Figure S3. Overexpression of the *E. faecalis* AcpA and PlsX proteins bypassed initiation of fatty acid synthesis in the *fabH* strain supplied with octanoic acid (C8:0). **A**. Growth of the *E. faecalis* Δ*fabH* strain overexpressing both AcpA and PlsX in the presence of octanoic acid on M17 medium. **B**. Synthesis of phospholipid fatty acyl chains by *E. faecalis* Δ*fabH* strain with overexpression of both AcpA and PlsX in the presence of octanoic acid. The numbers above the lanes are the radioactive label incorporation values relative to the value (100) for the wild-type strain cultured with 1 mM octanoic acid. **C**. Effects of overexpressing both cognate AcpA and PlsX on incorporation and elongation of [1-^{14}C]octanoic acid by the *E. faecalis* Δ*fabH* strain. The numbers above the lanes are the radioactive label incorporation values relative to the value (100) for the Δ*fabH* strain expressing only *L. lactis* PlsX.

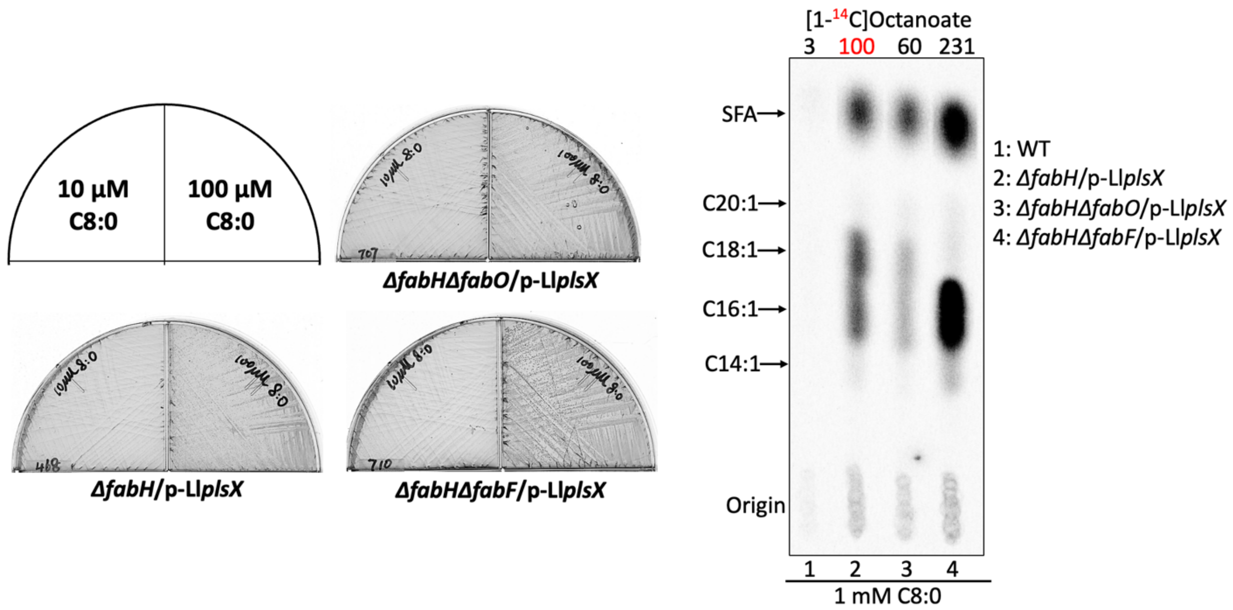

Fig. S4. The *E. faecalis* Δ*fabH* Δ*fabF* strain with *L. lactis* PlsX expression was more efficient in incorporation of octanoic acid than the Δ*fabH* Δ*fabF* strain with *L. lactis* PlsX expression (right panel) indicating that FabO is superior to FabF in elongation of octanoic acid. Note that 100 μM octanoate was needed for growth (left panel) with either FabO or FabF.

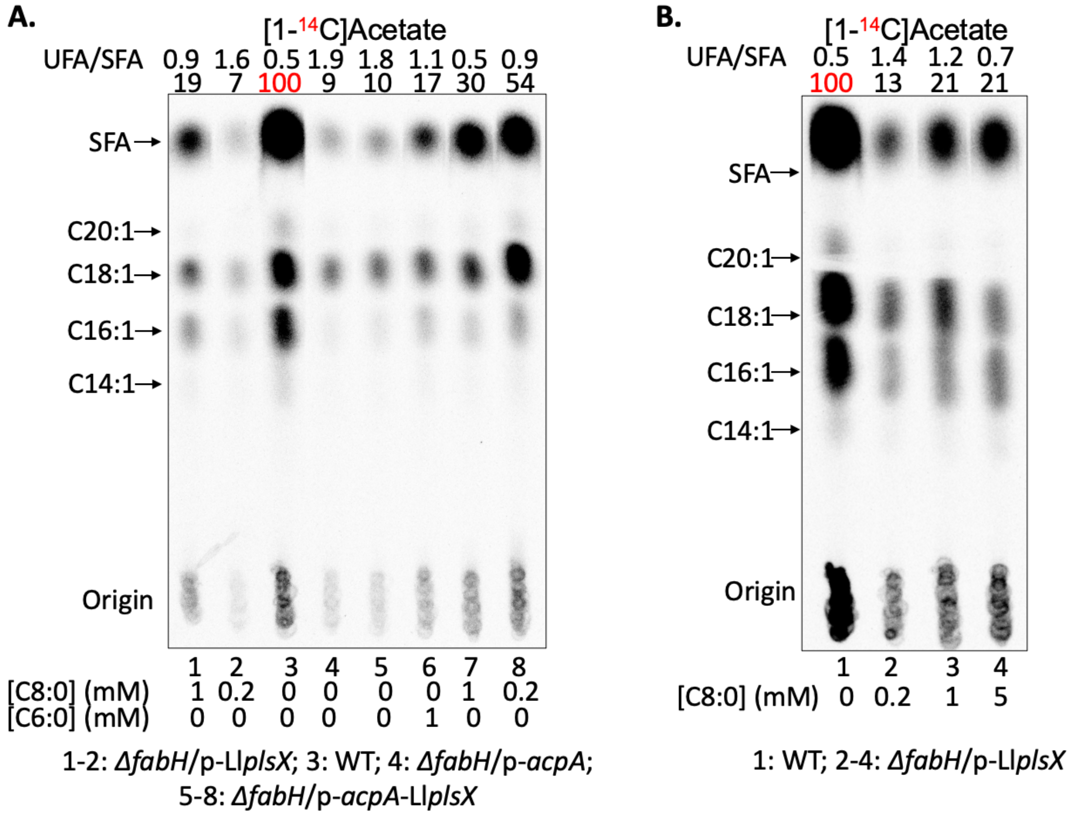

Figure S5. Membrane phospholipid unsaturation was increased by incorporation and elongation of octanoic acid (C8:0) in *E. faecalis* strains deficient in initiation of fatty acid synthesis. **A**. Synthesis of phospholipid fatty acyl chains by the *E. faecalis* Δ*fabH* strain with expression of *L. lactis* PlsX in the presence of octanoic acid supplied at 0.2 or 1 mM. **B**. Synthesis of phospholipid fatty acyl chains by the *E. faecalis* Δ*fabH* strain with expression of only *L. lactis* PlsX in the presence of octanoic acid supplied at 0.2, 1, or 5 mM. The numbers above the lanes show the membrane unsaturation and the radioactive label incorporation values relative to the value (100) for the wild-type strain without supplementation of octanoic acid. Note that lane 6 contained hexanoic acid (C6) in place of octanoic acid and like octanoic acid gave a 2- to 3-fold increase in [^{14}C]acetate incorporation over that of the Δ*fabH* strain. Note that unlike long chain acids, octanoic acid uptake does not repress *de novo* synthesis [31].

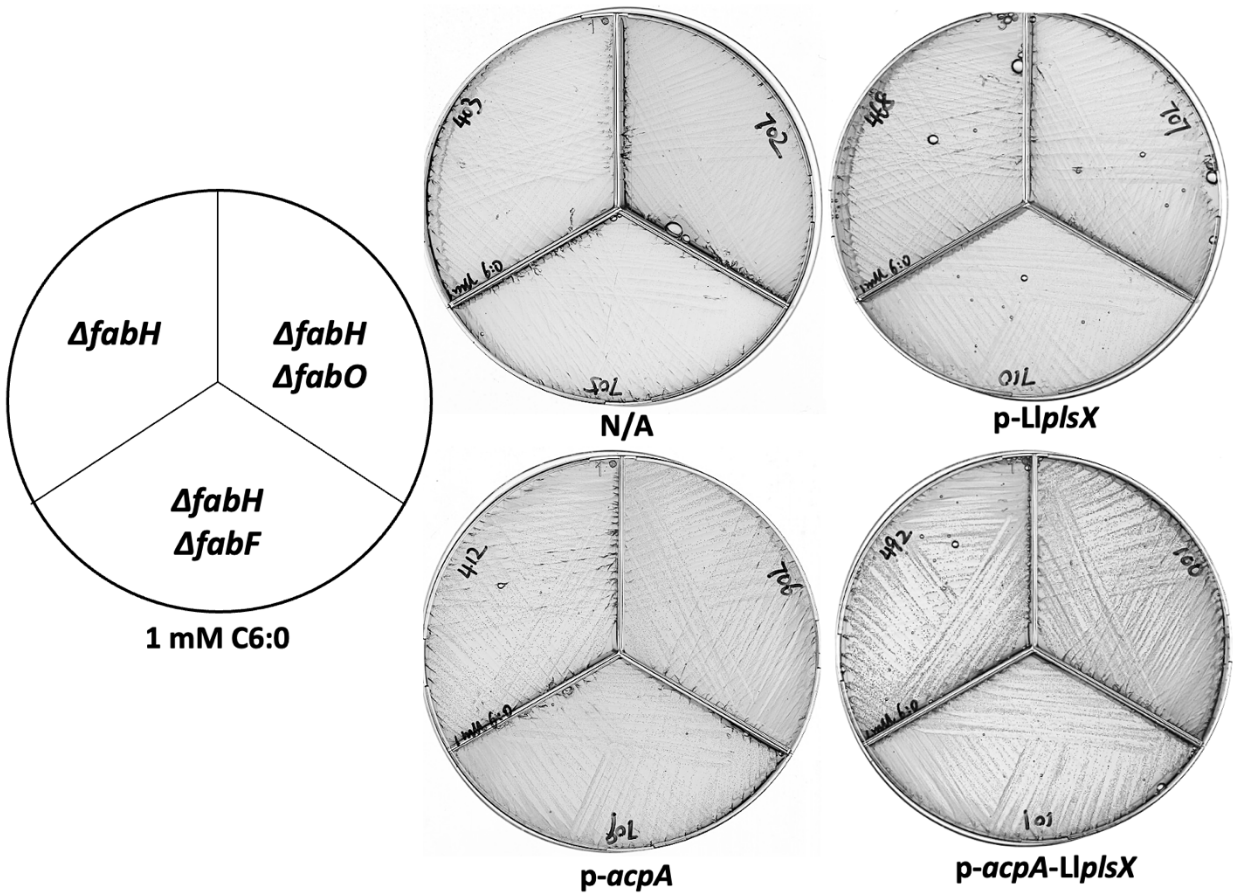

Fig. S6. Expression of *L. lactis* PlsX together with AcpA-overexpression aided bypass of the Δ*fabH* initiation defect in *E. faecalis* supplied with hexanoic acid (C6:0). Note however, that these manipulations also increased growth of the Δ*fabH* strain in 24 h incubations such that the growth observed cannot be ascribed to hexanoic acid supplementation.
